# Esophageal Clearance Patterns in Normal Older Adults as Documented with Videofluoroscopic Esophagram

**DOI:** 10.1155/2009/965062

**Published:** 2009-09-23

**Authors:** Janice Jou, Jason Radowsky, Ronald Gangnon, Elizabeth Sadowski, Stephanie Kays, Jacqueline Hind, Eric Gaumnitz, Andrew Taylor, JoAnne Robbins

**Affiliations:** ^1^Section of Gastroenterology and Hepatology, Department of Medicine, University of Wisconsin School of Medicine and Public Health, Madison, WI 53792, USA; ^2^Geriatric Research Education and Clinical Center, William S. Middleton Memorial Veterans Hospital, United States Department of Veteran Affairs, Madison, WI 53705, USA; ^3^Department of Biostatistics and Medical Informatics, University of Wisconsin School of Medicine and Public Health, Madison, WI 53792, USA; ^4^Department Radiology, University of Wisconsin School of Medicine and Pulic Health, Madison, WI 53792, USA

## Abstract

Normal esophageal bolus transport in asymptomatic healthy older adults has not been well defined, *potentially* leading to ambiguity in differentiating esophageal swallowing patterns of dysphagic and healthy individuals. This pilot study of 24 young (45–64 years) and old (65+years) men and women was designed to assess radiographic esophageal bolus movement patterns in healthy adults using videofluoroscopic recording. Healthy, asymptomatic adults underwent videofluoroscopic esophagram to evaluate for the presence of ineffective esophageal clearance, namely, intraesophageal stasis and intraesophageal reflux. Intraesophageal stasis and intraesophageal reflux were visualized radiographically in these normal subjects. Intraesophageal stasis occurred significantly more frequently with semisolid (96%) compared with liquid (16%) barium, suggesting that a variety of barium consistencies, as opposed to only the traditional fluids, would better define the spectrum of esophageal transport. Intraesophageal reflux was observed more frequently in older males than in their younger counterparts. The rates of intraesophageal stasis and intraesophageal reflux were potentially high given that successive bolus presentations were spaced 10 seconds apart. These findings suggest a need for a more comprehensive definition regarding the range of normal esophageal bolus transport to (a) prevent misdiagnosis of dysphagia and (b) to enhance generalization to functional eating, which involves solid foods in addition to liquids.

## 1. Introduction

Swallowing complaints are common presenting symptoms to general practitioners in the adult population. Surveys report that 30–40% of men and women between 50 and 79 years of age have an oropharyngeal or esophageal swallowing complaint [1, 2]. When these patients present to practitioners with symptoms of dysphagia, esophagrams often are used to evaluate for the presence of structural pathology or abnormal motility [3, 4]. Other diagnostic tests used in the evaluation of dysphagia include manometry, multichannel intraluminal impedance and endoscopy. 

The esophagram has become a common diagnostic procedure used for the evaluation of dysphagia given that the study is minimally invasive and clinically valuable in assessing esophageal dynamics relative to bolus transport [5] including reflux, tertiary contractions, other dysmotility abnormalities, and retention of barium in the esophagus. The test is considered abnormal when any of these findings are observed. For evaluating esophageal function, an esophagram is considered superior to endoscopy due to the dynamic aspects of the esophagram being able to view the progression of a bolus through the esophagus [6]. 

Videofluoroscopic esophagram differs from the conventional esophagram in that it is a real-time radiologic imaging study. In a videofluoroscopic esophagram, continuous images are transmitted to a videofluoroscopic recorder, or more recently, a DVD recorder, and monitor and can be reviewed on tape after the study is completed. Additionally, the use of a carefully selected and standardized range of stimuli representing key material properties in the protocol for the videofluoroscopic esophagram may elucidate more information about the interactions between swallowing physiology, biomechanics and the movement of boluses in the esophagus [7].

While extensively studied by manometry [8–10] and scintigraphy [11], the radiologic esophageal transport characteristics of older normal individuals have not been well defined. The primary objective of this pilot study is to document esophageal swallowing patterns in healthy normal older adults with fluoroscopic recording of esophageal bolus transport and to quantify the frequency of occurrence and significance of esophageal findings, including intraesophageal stasis (IES) and intraesophageal reflux (IER) [12, 13]. The current assumption in clinical practice is that the presence of IES and IER on esophagram is an abnormal finding, [14, 15] and thus, these entities are responsible for symptoms in symptomatic or dysphagic patients [16, 17]. However, we have noticed these types of bolus transport patterns occuring in normal individuals. At present, the significance and utility of reporting inefficiencies in esophageal transport to differentiate abnormal from normal patients remains limited.

## 2. Materials and Methods

### 2.1. Study Population and Design

Adult subjects were recruited on a volunteer basis from the community of Madison, WI, US, and its surrounding area. A total of 24 subjects (six females 45–64, six males 45–64, six females 65 and older, and six males 65 and older) gave informed consent and were subsequently enrolled in the study protocol. This study was conducted with the approval of the Institutional Review Board of the University of Wisconsin Health Sciences Center and the Research and Development Committee of the Williams S. Middleton Memorial Veterans Hospital.

All subjects were screened by a physician verbally prior to enrollment. Exclusion criteria included report of swallowing complaints, coughing while eating, gastroesophageal reflux, use of histamine blockers or proton-pump inhibitors, difficulty swallowing pills, coronary artery disease, diabetes mellitus requiring insulin, thyroid disease, or spinal surgery.

### 2.2. Recorded Fluoroscopic Esophagram

All enrolled subjects underwent an esophagram using videofluoroscopy (Siemens Sireskop Axiom, Panasonic SVHS AG-7350). In order to decrease radiation and to view as much of the esophagus as possible without moving the fluoroscope, the image intensifier grid was removed, the frames per second (fps) were reduced to 12.5 fps, and the field of view was changed to 16 cm. Initially, subjects were imaged while swallowing, including the oropharynx, using a sip of thin liquid barium from a cup to rule out oropharyngeal aspiration. If oropharyngeal aspiration was absent, the subjects proceeded through the remainder of the esophageal protocol. No patients were excluded from completing the protocol due to aspiration by this screening method. 

In an upright position, boluses were administered in the following order: three 10 mL of Varibar Thin Liquid barium (E-Z-EM, Inc.) via a 3oz pill cup, three 10 mL Varibar Pudding (semisolid) barium (E-Z-EM, Inc.) by teaspoon, and a 13 mm barium pill (E-Z-EM, Inc.) with a self-controlled water wash via a 3oz pill cup. The subjects were then placed in a right anterior oblique prone position and the following boluses were administered in the following order: three 10 mL Varibar Thin Liquid barium via 60 ml catheter syringe and three 10 mL Varibar Pudding (semisolid) barium via teaspoon. The patients were asked to swallow once and only once for each bolus given. Compliance was insured by the study personnel conducting the protocol. A bolus was repeated if a double swallow occurred. Prior to initiation of the next bolus, the subject was prompted to inform the study staff of his/her readiness for the next bolus. Dry swallows in between boluses were not evaluated. There was a time interval of at least 10 seconds between each bolus given.

### 2.3. Operational Definitions

**Figure 1:**
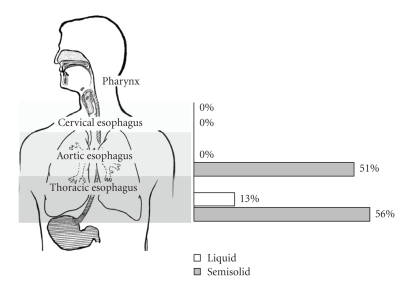
Regions of the esophagus defined by anatomic location and percentage of IES occurring with liquid and semisolid barium in the upright position.

Each videofluoroscopic esophagram was assessed for the presence and location of stasis or reflux (IES, IER, gastroesophageal reflux). The esophagus, as visualized fluoroscopically, was anatomically divided into three sections: (1) cervical esophagus—proximal to the clavicles, (2) aortic esophagus - from the clavicles distal to the aortic arch, (3) thoracic esophagus—from the bottom of the aortic arch to the lower esophageal sphincter (LES). (Figure 1). 

**Figure 2:**
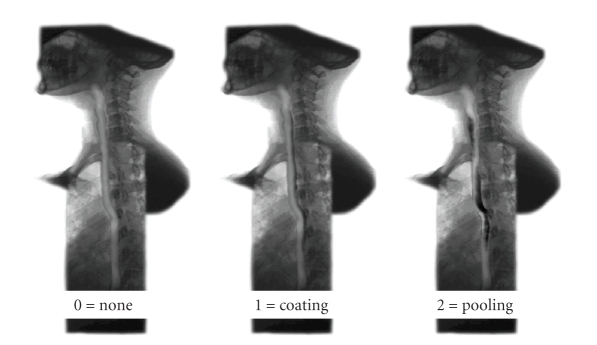
Grading of stasis of barium: the absence of IES was graded a 0, a coating of barium or a minimal amount of barium retention was scored as 1, and stasis with retained barium that completely filled the lumen of the esophagus was graded as 2.

Intraesophageal stasis (IES) occurred when any portion of the barium bolus failed to pass through the LES after completion of the initial swallow and coaptive primary peristaltic wave. The amount of barium retention was quantified on a scale of 0 to 2. The absence of IES was graded a 0, a coating of barium or a minimal amount of barium retention was scored as 1, and stasis with retained barium that completely filled the lumen of the esophagus was graded as 2 (Figure 2).

Intraesophageal reflux (IER) was present if any portion of the barium bolus traveled cephalad immediately after initial descent to a more proximal anatomic division of the esophagus prior to passing through the LES during the initial swallow (i.e., movement from the thoracic esophagus to the aortic esophagus). If dry swallows induced IER after administration of a bolus, the movement as a result of a dry swallow was ignored. Thus, a swallow with any element of IER was considered to exhibit IES as well. Gastroesophageal reflux was defined as any bolus that initially passes across the LES but travels cephalad back across the LES. The boluses with varying consistencies described above were administered in the supine and oblique positions. All boluses were confirmed to be cleared from the esophagus prior to initiation of the next bolus in the protocol.

### 2.4. Statistical Analysis

Repeated measures logistic regression models were used to determine the impact of age, sex, bolus type and position on intraesophageal stasis and reflux. Robust variance estimates were used to account for correlation between observations on the same subject. Age-sex and bolus type-position interactions were included in the models. Similar models were considered for intraesophageal stasis in each location (cervical, aortic, thoracic). Analyses were conducted using Proc Genmod in SAS (SAS Institute, Cary, NC, USA). A *P*-value of .05 was regarded as statistically significant.

## 3. Results

### 3.1. Reproducibility and Reliability

Two judges graded 20 swallows from multiple subjects chosen at random to determine intraobserver and interobserver reproducibility and reliability. There was 100% intraobserver reproducibility and reliability. In regard to interobserver reliability, there were 95% agreement for presence of stasis, 90% agreement for location of stasis, 80% agreement for presence of IER, and 93% agreement for the location of this reflux.

### 3.2. Bolus-Position Comparison

**Figure 3:**
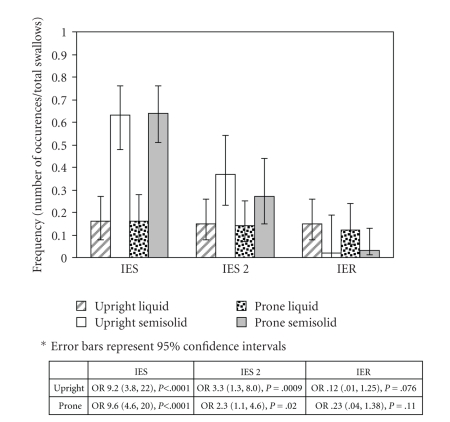
Frequency of IES, IES Grade 2 (IES 2) and IER in swallows of liquid and semisolid barium in the prone and upright positions.

IES was observed on at least one swallow in 96% of this healthy cohort (24/25), with the more severe IES Grade 2 occurring in 76% (19/25). IES occurred significantly more frequently with swallows of semisolid barium (63% upright, 64% prone) than with swallows of liquid barium (16% upright, 16% prone). Furthermore, IES Grade 2 (severe stasis) was significantly more common with semisolid (37% upright, 27% prone) than with liquid (15% upright, 14% prone) (Figure 3). 

In contrast, IER was observed on at least one swallow in 60% of the subjects (15/25). IER occurred more frequently with liquid (15% upright, 12% prone) than with semisolid (2% upright, 3% prone) (Figure 3). 

One female subject displayed stasis of the barium pill when the pill became lodged in the cervical esophagus in the upright position. The pill subsequently cleared the esophagus with multiple swallows completed during the remainder of the esophagram protocol. There was no reflux of the barium pill in any of the subjects enrolled. The rates of IES and IER did not vary significantly with subject position (*P* ≥ .24 for all comparisons).

### 3.3. Age-Gender Comparison

IES Stage 2 (severe stasis) occurred more frequently in older men (35%) than in younger men, although the difference was not statistically significant (13%, OR 3.7 (0.8, 17), *P* = .10); IES Stage 2 occurred at similar rates in older women (22%) and younger women (21%) (OR 1.1 (0.3, 4.0), *P* = .93). 

**Figure 4:**
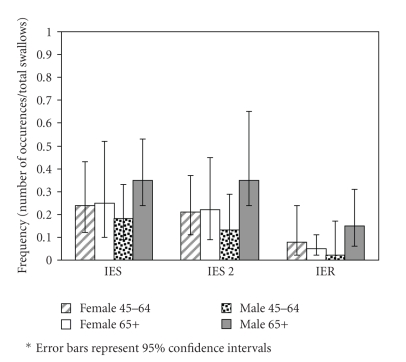
Frequency of IES, IES Grade 2 (IES 2) and IER in swallows of males and females ages 45–64 and 65 and older.

IER occurred more frequently in older men (15%) than in younger men (2%) (OR 8.1 (0.9, 73), *P* = .06) or older women (5%) (OR 3.2 (0.9, 12), *P* = .08). IER rates did not differ significantly between older and younger women (OR 0.6 (0.2, 2.4), *P* = .47) or between younger men and younger women (OR 0.2 (0.03, 2.2), *P* = .20) (Figure 4).

### 3.4. Location of IES Comparison

Interestingly, there was no IES with liquid or semisolid in the cervical region and no IES with liquid in the aortic region with any swallow in the upright position (Figure 1). In the upright position, IES occurred significantly more frequently with semisolid as compared with liquid in the thoracic location (OR 8.41 (3.24, 21.8), *P* < .0001). In the prone position, IES occurred significantly more frequently with semisolid as compared with liquid in both the aortic (OR 9.41 (4.53, 19.57), *P* < .0001) and thoracic locations (OR 8.41 (4.1, 17.3), *P* < .0001).

## 4. Discussion

Results from this pilot study provide evidence that a surprisingly high number of asymptomatic healthy adult subjects exhibit esophageal bolus movement patterns that are often considered to be abnormal clinically, with 96% of this sample exhibiting IES and 60% exhibiting IER at least once during a videofluoroscopic esophagram. The rates of IES and IER were potentially higher given that the time between swallows was 10 seconds rather than 20–30 seconds described in prior protocols investigating dysphagia [18]. Additionally, the power to detect differences between age and gender groups was limited given the modest sample size of this cohort. However, these data provide a framework for further studies of a larger magnitude to assess for possible differences in swallowing of normal order individuals

Healthy individuals are by definition not symptomatic, although they might display inefficient esophageal bolus transport as measured with radiography. Normal subjects may have variation in esophageal bolus transport displayed by the occurrence of inefficient bolus progression; however it may be at a less frequent rate than in dysphagic persons thus highlighting the correlation of the frequency of inefficient bolus progression to symptoms. Symptomatic patients also may demonstrate increased sensitivity to distension of the esophagus or chronicity of abnormal esophageal bolus transport.

The rates of IES with liquid barium (16%) were similar to those described in normal individuals by Imam et al. [19] who reported a 10% rate of stasis with liquid barium. However, these results are in contrast to those reported by Ott et al. [18], in which no normal controls had ineffective esophageal bolus transport as viewed with fluoroscopy. There are probably multiple factors that lead to the different results from the present compared with Ott et al. [18]: the use of different barium materials comprising only liquids; the systematic swallowing protocol followed for the videofluoroscopic esophagram, including the simultaneous performance of manometry in the upright position only; and the use of broad criteria for defining abnormal esophageal swallowing (a disruption in the peristaltic wave) [20]. 

Our results also differed from the results in normal individuals seen by Tutuian et al., who reported on combined results of multichannel intraluminal impedance and manometry in normal individuals [21]. Videofluoroscopic esophagram was not used in the protocol. In the study by Tutuian et al., more than 93% of normal individuals had at least 80% complete liquid or at least 70% complete viscous bolus transit. These results were in a younger population with a mean age of 38 (range 21–72 years) whereas our cohort was of older adults aged 45 and older. (REF- Tutuian et al.)

Gravity is thought to play a significant role in aiding the swallow mechanism for those with dysphagia [22]. In our cohort of normal individuals, gravity did not affect the occurrence of IES or IER. Notably, when IES did occur in the cervical region, it occurred in the prone position and was more likely manifest with semisolid as compared to liquid. Therefore, gravity may influence swallowing to a greater degree in those who do display IES with semisolid barium, and may play a role in determining the location where the bolus arrests in its path. Such deviations in esophageal bolus clearance patterns may be important markers for identifying individuals potentially susceptible to developing symptoms in response to minor insults to the swallowing mechanism. 

The only gender or age-related effect observed was a trend of increased IER in males age 65 and older. This is an important consideration as IER and IES could be risk factors for the development of esophagitis and aspiration pneumonia. Additionally, significantly more stasis emerged in the aortic esophagus with semisolid as compared to liquid barium. The finding of stasis at the level of the aortic arch can also be accounted for by the presence of a transition zone where there is a delay and/or spatial gap between the terminus of the proximal esophageal contraction and initiation of the distal esophageal contraction as described by Ghosh et al. [23]. Additionally, the natural tendency for esophageal contents to remain in the aortic esophagus might be worsened if there was any ectasia or tortuosity at the aortic arch. Cardiac disease, which may be associated with aortic arch prominence, is the second most common associated comorbidity with aspiration pneumonia [24]. The next step would be to assess if pathology affecting aortic size affects barium retention and if patients are indeed more symptomatic when boluses are retained around the aortic arch. 

The short interval between swallows is a potential limitation of the study because of the second bolus arriving prior to the conclusion of the refractory period from the first. However, given the rapid rate of eating in our fast-paced society, it also could be considered a more accurate representation of mealtime behavior. Patient positioning prevents the comparison of outcomes with some esophageal motility studies in that patients in this study were evaluated in the prone position (standard for videofluoroscopic studies) while traditional esophageal motility studies are completed with the patient in a supine position.

## 5. Conclusions

In our normal cohort, semisolid boluses elicited significantly more stasis than liquid boluses. This suggests that a variety of barium product consistencies simulating food-like viscosities need to be used, in addition to traditional fluids, to fully evaluate esophageal transport with the videofluoroscopic esophagram. Our protocol included observations of esophageal motility with semisolid barium which has not been documented previously. Modification of the esophagram protocol may increase the sensitivity of detecting abnormal esophageal motility in those patients who are symptomatic. 

Furthermore, the definitions used at present to define normal versus abnormal esophagram characteristics must be questioned. Ineffective bolus transport is seen in normal individuals, and it remains uncertain how these patterns differ in dysphagic patients. Given the modest sample size, additional studies are required to further explore the findings in this pilot study. Future efforts will provide a normative database for comparison with dysphagic patients and further define the range of normal esophageal swallowing patterns. Such information will be essential in minimizing false positives, finding the practitioners' tendencies toward the excessive testing in health care and the high costs associated with medical diagnostic care provision in later life.
